# Evaluation of the maxillary midline, curve of the upper lip, smile line and tooth shape: a prospective study of 140 Caucasian patients

**DOI:** 10.1186/s12903-020-1031-y

**Published:** 2020-02-06

**Authors:** María Melo, Javier Ata-Ali, Fadi Ata-Ali, Marco Bulsei, Perluigi Grella, Teresa Cobo, José María Martínez-González

**Affiliations:** 1 Valencia University Medical and Dental School, University of Valencia, Valencia, Spain; 2 Department of Dentistry, Universidad Europea de Valencia. Faculty of Health Sciences, Valencia, Spain; 3 Dentist, Public Dental Health Service, Conselleria de Sanitat Universal i Salut Pública, Generalitat Valenciana, Valencia, Spain; 4 Department of Surgery and Medical-Surgical Specialities, Area of Orthodontics, University Medical and Dental School. University of Oviedo (Spain). Instituto Asturiano de Odontologia, Oviedo, Spain; 5 Department of Medicine and Oral Surgery, Faculty of Dentistry, Complutense University of Madrid, Madrid, Spain

**Keywords:** Smile line, Dental aesthetics, Tooth shape, Dental midline, Facial aesthetics

## Abstract

**Background:**

A number of reference patterns such as the interincisal line, curve of the upper lip, width of the smile or shape of the teeth have been studied in different populations. Determining the frequency of different smile aesthetic parameters in a European Caucasian population and exploring possible gender differences is important in order to obtain predictable treatment outcomes.

**Methods:**

Photographs were obtained under resting and forced smiling conditions in 140 individuals (70 males and 70 females) with a mean age of 20.1 ± 4.3 years. Different variables were recorded, including the position of the maxillary interincisal midline in relation to the facial midline, the arc and width of the smile, and the shape of the teeth. The data were processed using the SPSS version 15.0 statistical package, with application of the chi-squared test and a confidence level of 95%. The statistical power was 80%, and the level of significance 5% (α = 0.05).

**Results:**

A total of 94.3% of the sample presented a maxillary interincisal midline coinciding with the facial midline, and 80% had a consonant smile line. The curve of the upper lip was upwards in 47.1% of the cases, followed by a straight curve in 41.4%. Most of the subjects (84.3%) presented a medium smile line with tooth exposure to the second premolar (61.4%). There were no significant differences between males and females.

**Conclusions:**

The integration of aesthetic criteria is needed in order to guarantee satisfactory and predictable dental treatment outcomes. There were no statistically significant differences between males and females. The maxillary interincisal midline coincided with the facial midline, with a consonant smile arc and a medium smile line, upward lip curve and oval tooth shape.

## Background

The ideal characteristics of a smile have been described by different authors [1, 2], though deviations from the classical aesthetic dimensions are found in the different studied populations [3–5]. Different methods are available for analysis of the smile, including photographs [6, 7], videos [8, 9] or the use of three-dimensional (3D) stereophotogrammetric images [10].

There are two types of smile: spontaneous and forced. The former is involuntary, with elevation of the lip induced by happiness, and can be taken to express genuine emotion [11]. Elevation of the lip is greater than in the case of a posed smile [12], which is reproducible and thus involves a reference position [13]. In facial analysis we have a number of horizontal reference lines (the bipupillary line and other lines parallel to the latter) and the vertical midline located by two anatomical reference points: the nasion and the filtrum [6]. The more perpendicular this line is to the bipupillary line, the greater the sensation of total harmony of the face [13]. The maxillary interincisal midline should coincide with the facial midline, and when this is not possible, it should lie parallel to the facial midline [14]. Kokich et al. reported that a variation between the facial line and the maxillary interincisal midline limited to 4 mm is not perceptible to either patients or dental professionals [15], though other authors consider that a smaller difference proves evident. This is the case of Sadrhaghighi et al. [16], who recorded a deviation of the maxillary interincisal midline with respect to the facial midline of between 1 and 3 mm. Pinho et al. [17] in turn found that orthodontists are able to detect any lack of coincidence of the two midlines, while prosthodontists detect no evident alterations until the difference reaches at least 2 mm. Zhang et al. [18] found that the side to which the maxillary interincisal midline is deviated (right or left) resulted in no significant differences in terms of perception of the deviation. In contrast, an inclined midline would be more evident, and therefore less acceptable [19]. However, a midline deviation of over 2 mm, an inclination of the midline of 3.5 degrees, and an inclination of the incisal plane of 2 degrees have been described as defining a scantly aesthetic smile in another study [20].

The arc of the smile can be defined as the relationship between the curve of the incisal margins of the upper incisors and canines and the curve of the lower lip in a posed smile, and may be consonant or non-consonant [12]. The curve of the upper lip can be divided into three categories according to the position of the corners of the mouth in relation to the center of the lower margin of the lip: upward, straight or downward [21]. The smile line in turn is classified as high if it exposes the entire clinical crown together with a continuous gingival band of variable size; medium if it exposes 75–100% of the clinical crown and only the interdental papilla; or low when the smile exposes less than 75% (three-quarters) of the clinical crown [22]. The shape of the maxillary central incisors is determined by their incisal-cervical height and maximum mesiodistal width. Marvouskoufis and Ritchie [23] reported that 86–90% of the studied population did not exhibit exactly the same dimensions in both contralateral teeth, while other authors found no statistically significant differences [24, 25], and any such differences were not relevant from the clinical perspective [3].

The present study was carried out to determine whether there are gender differences in a number of smile aesthetic parameters, with the purpose of facilitating the planning of multidisciplinary treatment. These parameters are coincidence of the maxillary interincisal midline with the facial midline, the arc of the smile, curve of the upper lip, line and width of the smile, and the shape of the upper central incisors. Our working hypothesis was that most of the population presents a maxillary midline centered with respect to the facial midline, a consonant arc of the smile, an upward curve of the upper lip, a medium smile line and ovoid teeth [3, 6, 26].

## Methods

In the present prospective study, photographs were obtained under resting and forced smiling conditions in 140 individuals with a mean age of 20.1 ± 4.3 years (range 18–30), at the Universidad Europea de Valencia (Valencia, Spain). The study was conducted in accordance with the Declaration of Helsinki, and was approved by the local Research Ethics Committee (Ref. CIPI/100/17).

All enrolled subjects were of Caucasian origin, with complete and fully erupted permanent dentition, and with no current or past orthodontic treatments. Before the start of the study, all individuals were fully informed on the nature of this study, read and signed a written consent form for inclusion, and for the analysis of the records obtained, that was approved by the University. Individuals with a history of orofacial trauma were excluded, as were those with anterior sector prostheses or dental implants, periodontal disease, incisal marginal wear, and caries with the loss of tooth material [4].

The photographs were obtained with a DSLR camera (Nikon, model D3100) using an 18–55 mm objective (Tokina) and a tripod (Canon Inc.) positioned at a distance of 40 cm from the study subject. The participants were positioned sitting in a chair placed 10 cm from the wall in order to avoid shadow effects, with the head relaxed and the gaze focused on an external point at eye level so that the Frankfort plane and the bipupillary line were parallel to the axis of the objective of the camera positioned in the same plane for obtaining the extraoral photographs [27]. The smile was photographed from the frontal position with the axis of the objective of the camera in the same plane as the occlusal plane. In obtaining the photographs, the authors always sought to maintain parallelism through the guiding reference lines in the visor of the digital camera, and a millimetered ruler was positioned on one side of the head of the patient to calibrate the real dimensions of the photographs on analyzing the images. In the frontal facial photographs, the focus point and center of the photograph were the intersection between the horizontal Frankfort line and the midline of the face, while for the intraoral photographs they were located between the incisal plane and the dental midline.

A single trained investigator (MM) recorded all the parameters. Adobe Photoshop CC2018 (Adobe Systems Incorporated Co.) and Adobe LightRoom 6 (Adobe Systems Incorporated Co.) were used by two investigators (PG and MB) to evaluate the characteristics of the smile. The images were examined independently by each examiner, and the results of the evaluated features were subsequently compared. In the event of disagreement, the image was again evaluated jointly to reach consensus. The level of agreement between the two authors was assessed based on the Cohen kappa statistic. The maxillary interincisal midline [19] was classified as either centered or deviated in relation to the facial midline, determined by the line from nasion to filtrum. The arc of the smile in turn was classified as either consonant and non-consonant, while the curve of the upper lip was classified as upward, straight or downward [12, 22]. Depending on the amount of tooth and gingiva exposed on smiling, this line was defined as high, medium or low [28, 29]. Smile width was determined according to the number of teeth exposed on smiling [30, 31]. The relationship between the shape and proportion of the central incisors (DP) was calculated using the formula DP% = maximum width (mm) / length (mm) × 100, classifying the teeth as triangular, ovoid or square [32].

### Statistical analysis

The data were entered on a spreadsheet for subsequent analysis using the SPSS version 15.0 statistical package. A descriptive study was made based on statistical frequency for each of the parameters in the global sample and stratified according to gender. Subject gender was defined as the fundamental independent variable of the study, with a view to determining the existence of sexual dimorphism associated to the mentioned parameters.

A bivariate analysis was carried out using the chi-squared test to assess the degree of association between the different parameters and patient gender, since it can be employed with data measurable on a nominal scale. Perfect coincidence between the observed and the expected frequencies received a value of 0. In contrast, large discrepancies between the frequencies resulted in a large value of the statistic, with consequent rejection of the null hypothesis. Accordingly, the critical region was located at the upper range of the chi-squared distribution with k-1 degrees of freedom. The Fisher exact test was used in those cases where the contingency tables presented many cells (> 33%) with low expected frequencies (< 5 cases). This allowed us to determine whether two dichotomic variables are related. The Mann-Whitney U-test is a nonparametric test applied to two independent samples. It was used to contrast the homogeneity of distribution of a variable between two independent groups, such as smile line according to gender. The study sample presented a statistical power of 80% with a confidence level of 95% in detecting statistically significant differences between proportions 50 and 65% in two groups (according to gender) using the chi-squared test. The level of significance was 5% (α = 0.05). The sample size was calculated from the following formula: Potency = $1-\beta ={F}_{gl, lambda,1-\alpha}\left({\aleph}_{crit}^2\right)$, where: df = degrees of freedom, lambda (noncentrality parameter) = w^2^ n, w (effect size) = $\sqrt{\sum \limits_{i=1}^m\frac{{\left({p}_{oi}-{p}_{1i}\right)}^2}{p_{oi}}}$, p_0i_, p_1i_ = the sample proportions, n = the sample size, α = critical probability level, ${\aleph}_{crit}^2$ = the critical distribution value ${\aleph}_{gl,1-\alpha}^2$ and F = cumulative distribution function for *ℵ*^2^ with the noncentrality parameter lambda.

## Results

Photographs corresponding to 140 individuals (70 males and 70 females) with a mean age of 20.1 ± 4.3 years were analyzed. Table 1 shows the statistical results referred to the associations between the study parameters and gender. None of the parameters showed significant differences in terms of gender. The kappa coefficient for interrater reliability was 0.81.

**Table 1:** Association between different study parameters and gender. Results of Chi^2^ test, Fisher test (Fis) and Mann-Whitney (MW). Maxillary Interincisal mildilne (IML), facialmidline (FML), arch of the smile (ArS), curve of the lip (CL), smile line (SL), width of the smile (WS), shape of the teeth (Sh)

	*p*-value
IML/FML	0,614 (Fis)
ArS	1000 (Chi^2^)
CL	0,315 (Chi^2^)
SL	0,135 (MW)
WS	0,951 (Chi^2^)
Sh	0,379 (Chi^2^)

### M**axillary interincisal midline** versus facial midline

**Fig. 1:**
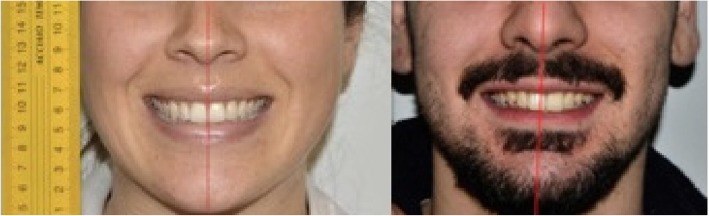
Coincidence of the maxillary interincisal midline with the facial midline (left), and absence of coincidence (right)

In the great majority of the subjects (94.3%; *n* = 132), the maxillary dental midline coincided with the facial midline, while 5.7% (*n* = 8) showed deviation (Fig. 1) (Table 2).

**Table 2:** Frequency and percentage of the interincisal midline versus facial midline in the sample according to gender

	GENDER					
	Total		Female		Male	
	N	%	N	%	N	%
Total	140	100,0%	70	100,0%	70	100,0%
Centered	132	94,3%	68	97,1%	64	91,4%
Deviated	8	5,7%	2	2,9%	6	8,6%

### Arc of the smile

**Fig. 2:**
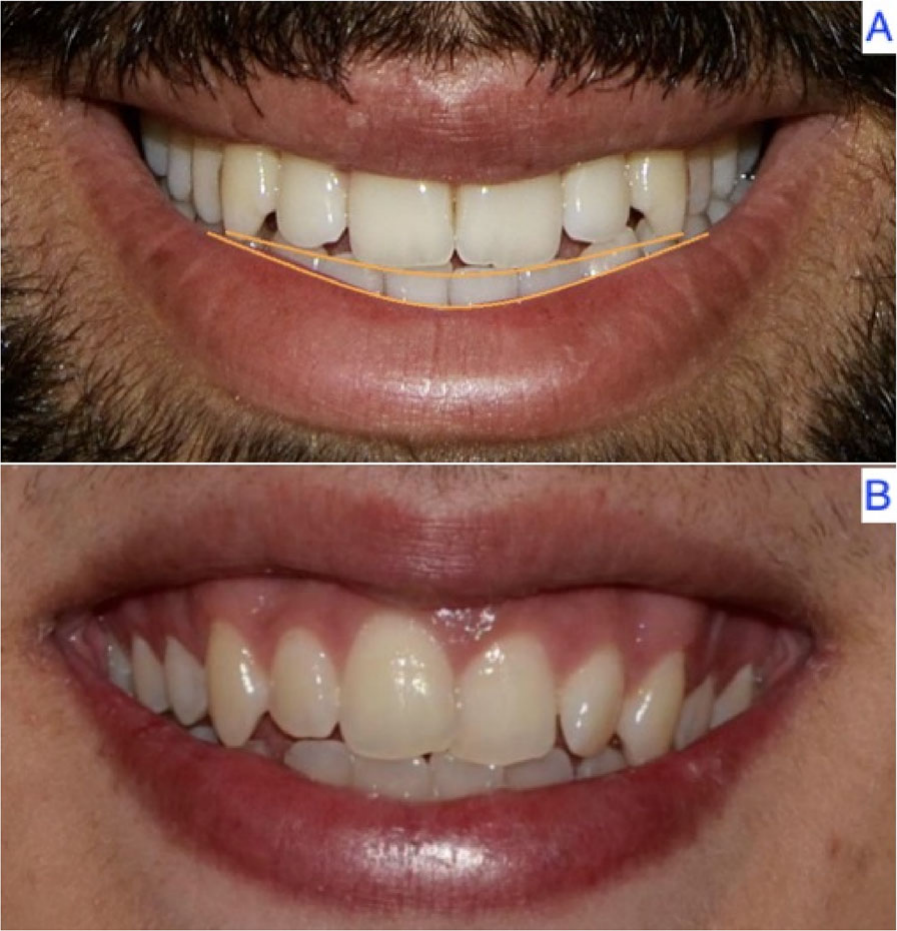
Consonant (**a**) and non-consonant arc of the smile (**b**), determined by the upper incisal line and the internal surface of the lower lip

Of the 140 individuals evaluated, 80% (*n* = 112) presented a consonant arc of the smile, while the remaining 20% (*n* = 28) showed a non-consonant arc. There were no statistically significant differences between males and females (*p* = 1) (Fig. 2) (Table 3).

**Table 3:** Frequency and percentage of the arc of the smile in the sample according to gender

	GENDER					
	Total		Female		Male	
	N	%	N	%	N	%
Total	140	100,0%	70	100,0%	70	100,0%
Centered	112	80,0%	56	80,0%	56	80,0%
Deviated	28	20,0%	14	20,0%	14	20,0%

### Curve of the upper lip

**Fig. 3:**
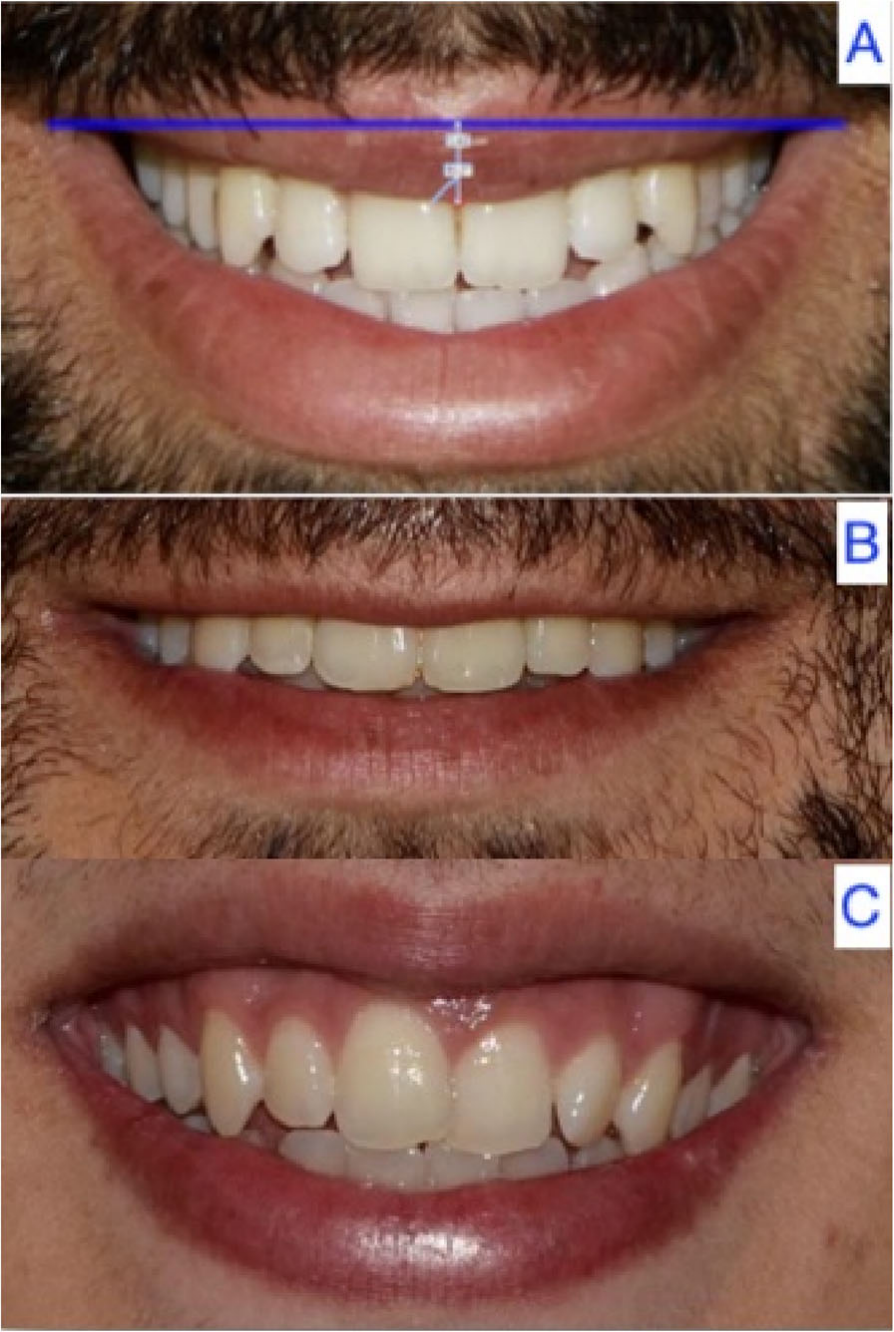
Upward (**a**), straight (**b**) and downward curve of the upper lip (**c**)

A total of 47.1% of the patients (*n* = 66) had an upward lip curve, 41.4% (*n* = 58) had a straight curve, and 11.4% (*n* = 16) presented a downward upper lip curve (*p* = 0.315) (Fig. 3) (Table 4).

**Table 4:** Curve of the upper lip in the sample according to gender

	GENDER					
	Total		Female		Male	
	N	%	N	%	N	%
Total	140	100,0%	70	100,0%	70	100,0%
Upward	66	47,1%	36	51,4%	30	42,9%
Straight	58	41,4%	30	42,9%	28	40,0%
Downward	16	11,4%	4	5,7%	12	17,1

### Smile line

**Fig. 4:**
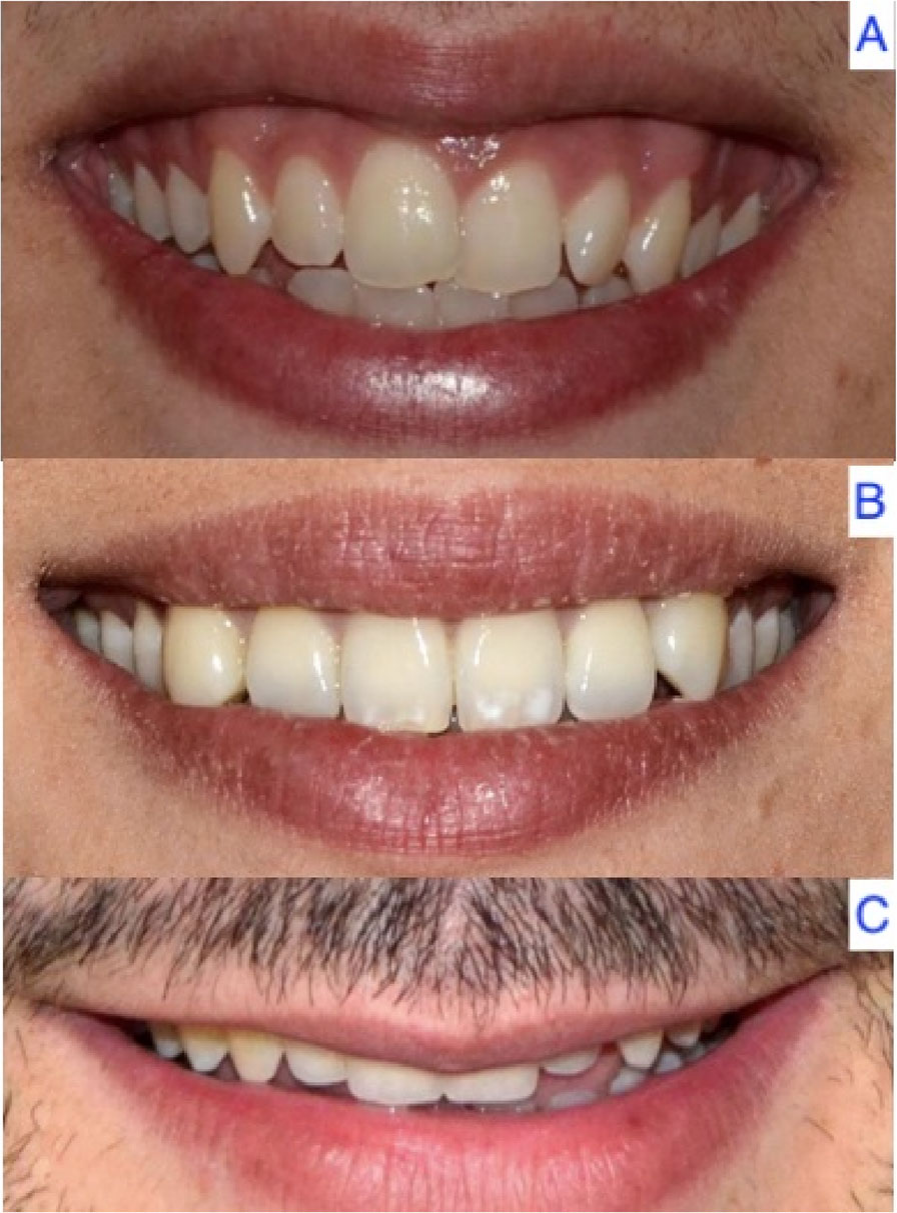
High (**a**), medium (**b**) and low smile line (**c**)

Most of the patients (84.3%; *n* = 118) presented a medium smile line, 8.6% (*n* = 12) a low smile line, and 7.1% (*n* = 10) a high smile line. According to gender, females more often presented a high smile line compared with males (11.4% versus 2.9%, respectively). In contrast, males showed a higher frequency of low smile lines (11.4% versus 5.7%) (*p* = 0.135) (Fig. 4) (Table 5).

**Table 5:** Smile line in the sample according to gender

	GENDER					
	Total		Female		Male	
	N	%	N	%	N	%
Total	140	100,0%	70	100,0%	70	100,0%
Medium	118	84,3%	58	82,9%	60	85,7%
Low	12	8,6%	4	5,7%	8	11,4%
High	10	7,1%	8	11,4%	2	2,9%

### Width of the smile

**Fig. 5:**
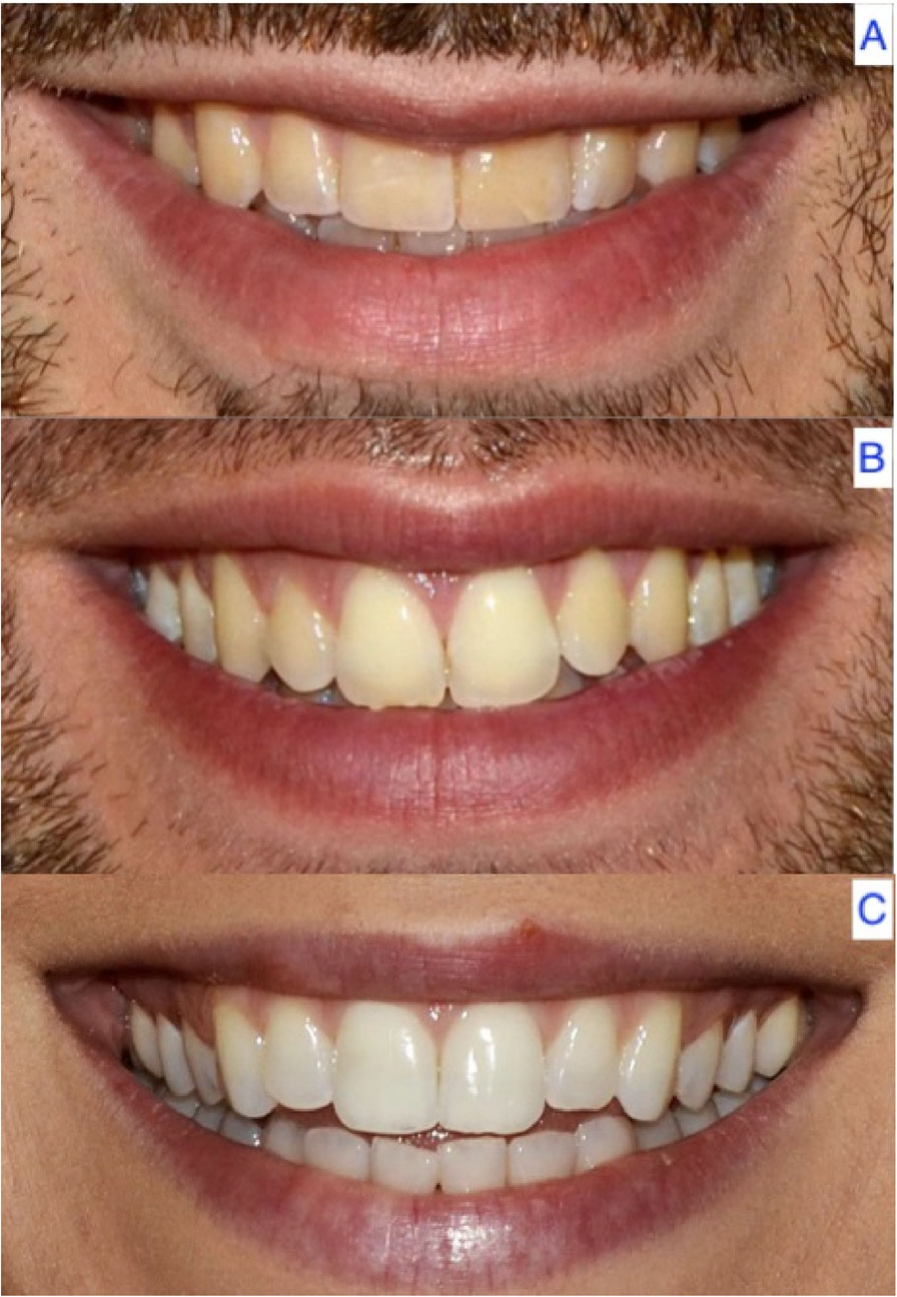
Smile width with exposure to the first premolar (**a**), second premolar (**b**) and first molar (**c**)

Of the 140 patients evaluated, 61.4% (*n* = 86) showed tooth exposure to the second premolar, 20% (*n* = 28) to the first molar, and 18.6% (*n* = 26) to the first premolar (*p* = 0.951) (Fig. 5) (Table 6).

**Table 6:** Width of the smile in the sample according to gender

	GENDER					
	Total		Female		Male	
	N	%	N	%	N	%
Total	140	100,0%	70	100,0%	70	100,0%
Second premolar	86	61,4%	44	62,9%	42	60,0%
First molar	28	20,0%	14	20,0%	14	20,0%
First premolar	26	18,6%	12	17,1%	14	20,0%

### Shape of the teeth

**Fig. 6:**
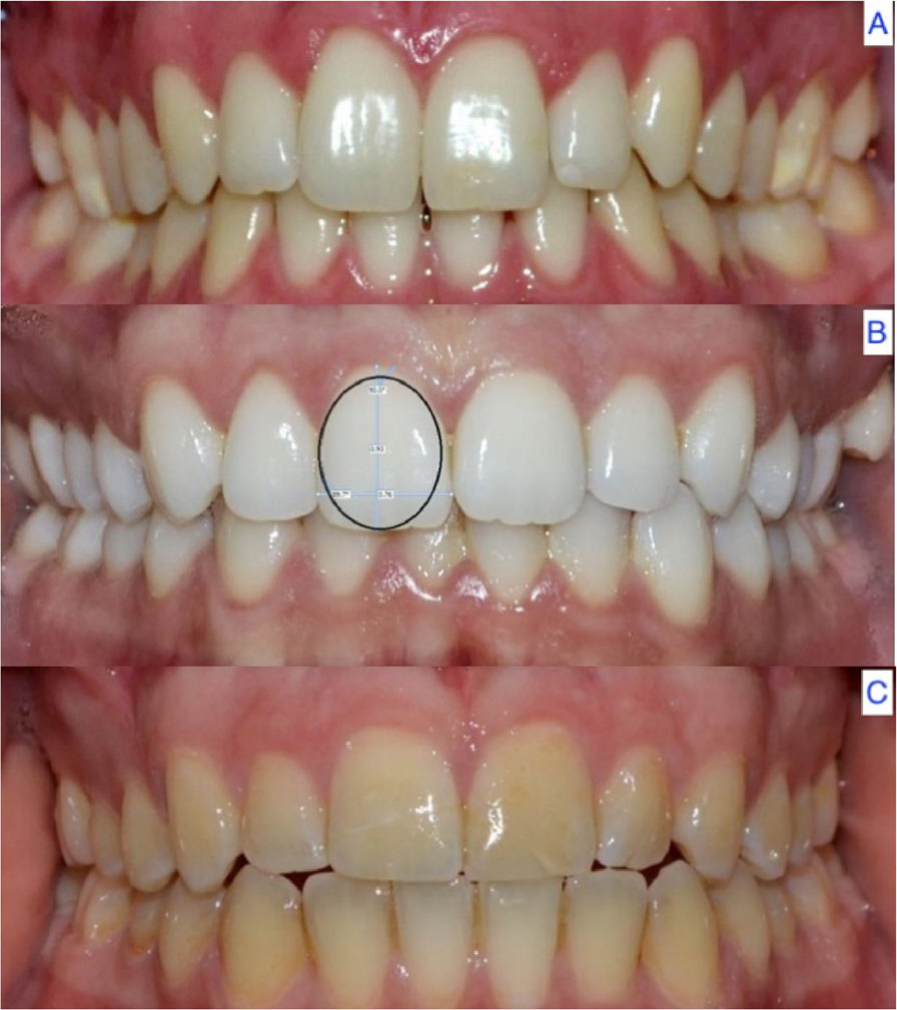
Tooth shapes: triangular (**a**), oval (**b**) and square (**c**)

A total of 62.9% of the subjects (*n* = 88) presented an oval tooth shape, 22.9% (*n* = 32) a square shape, and 14.3% (*n* = 20) a triangular tooth shape (*p* = 0.379) (Fig. 6) (Table 7).

**Table 7:** Frequency and percentage of the tooth shape according to gender

	GENDER					
	Total		Female		Male	
	N	%	N	%	N	%
Total	140	100,0%	70	100,0%	70	100,0%
Ovoid	88	62,9%	46	65,7%	42	60,0%
Square	32	22,9%	18	25,7%	14	20,0%
Triangular	20	14,3%	6	8,6%	14	20,0%

## Discussion

The present study was carried out to explore possible gender differences in certain aesthetic parameters, and their frequency in the population considered. Most of the patients were seen to have a maxillary interincisal midline coinciding with the facial midline, a consonant smile arc, and a medium smile line. The null hypothesis that there are no differences in these parameters on comparing them with other studies populations was therefore met.

Ethnicity and age are key conditioning factors of smile characteristics [22, 33], and for this reason they were clearly defined in our inclusion and exclusion criteria in order to secure a homogeneous sample of subjects. Our series showed a predominance of medium smile lines (84.3%), in coincidence with the observations of other authors [19, 22, 31, 33].

The ideal smile line is that in which the maxillary interincisal midline is centered with respect to the facial midline, and the teeth are fully exposed along with about 1 mm of gingival tissue. Accordingly, a smile that exposes no more than 2–3 mm is regarded as aesthetically pleasant, while the exposure of over 3 mm of gingival tissue is regarded as scantly attractive by most patients [27]. From the perspective of orthodontists, Dindaroğlu et al. reported that the amount of tooth exposed in the smile is decisive for regarding a smile as aesthetically pleasant (*p* = 0.018) [9]. Del Monte et al. [34] found that there should be 0.4 mm of gingival display when smiling. Asymmetry during smiling could be considered clinically as a dental occlusal cant or maxillary skeletal asymmetry [10], and it is crucial to know the underlying cause through careful diagnostic evaluation, in order to define the treatment best suited to each individual case.

In our sample, a high smile was more common among females than in males, while a low smile line was more prevalent among males - this being consistent with the data found in the literature [29, 33, 35]. In our study, 84.3% of the sample presented a medium smile line, 8.6% a low smile line and 7.1% a high smile line. The height of the smile line decreases with age as a result of the loss of supporting tissues. A high smile line poses a challenge for aesthetic restoration of the anterior sector [28], and a combined multidisciplinary management approach may be required [36, 37].

With regard to the arc of the smile, our results show a greater frequency of cases with a consonant arc (80%). This is in contradiction to the results published by Maulik and Nanda [31] – the reason probably being the use of different inclusion criteria, since the mentioned authors did not specify the ethnic composition of their sample. However, our findings do coincide with those of other authors [19, 22, 33]. A parallel smile arc is the most aesthetic and attractive presentation, followed by a straight arc, while an inverted arc is considered to be scantly aesthetic [33]. Lombardi and Desai et al. reported that the arc of the smile is related to the age of the individual [22, 38]. In youth, the central incisors are more prominent, generating a curve that is parallel and consonant with the lower lip, while in older individuals the curve tends to straighten as a result of wear.

The facial midline and maxillary interincisal midline were seen to be coincident in 94.3% of the studied subjects. These results are comparable to those of other publications [29, 39]. A coincident midline is the most prevalent presentation in nature and must be respected in prosthetic dental treatment in order to ensure an aesthetic outcome [33, 40]. Silva et al. [7] found the threshold for perceiving a deviation of the maxillary interincisal midline with respect to the facial midline to be 2 mm, in coincidence with the observations of other authors [16, 18]. Orthodontists are able to detect alterations of the maxillary interincisal midline with respect to the facial midline of as small as 1 mm. Parrini et al. [41] conducted a systematic review of smile perception among laypersons, and found a mean deviation of 2.38 mm to be acceptable.

In those cases where a coincident midline is not possible, the midline between the central incisors must be parallel to the facial midline [29]. The greater the discrepancy between these lines, the greater the asymmetry of the patient smile, and this proves evident even for an inexpert observer [42]. An incorrect inclination of the maxillary interincisal midline can be immediately recognized by any observer as scantly aesthetic, being less attractive than a lateral deviation of the maxillary interincisal midline with respect to the facial midline [13]. Thomas et al. found angulation of the maxillary interincisal midline to have a tolerance limits of 10 ± 6 degrees in laypersons [43].

With regard to tooth shape, our global sample showed a predominance of oval shaped teeth (62.9%), in concordance with the observations of other authors [44, 45]. It is important to know the aesthetic preferences of the population when designing rehabilitation. Thus, according to Alvarez-Alvarez et al. [46], both dentists and laypersons consider the proportion of 85% for the central incisor and 80% for the lateral incisor and canine to be the most aesthetic scenario.

The bivariate analysis revealed no statistically significant differences according to gender, in coincidence with data found in the literature [33]. However, Brunetto et al. did record a significant gender difference in that males tended to show a more triangular dental shape versus a more square shape in females [45]. This significant gender difference can probably be explained by the fact that the mentioned authors analyzed 433 Brazilian patients between 15 and 30 years of age, i.e., their sample characteristics were different from our own. In the study published by Anderson et al. [47], square-round incisors were preferred for males by all the groups surveyed (***p*** ≤ 0.042). In contrast, the shape regarded by orthodontist as most aesthetically pleasing for females was round and square-round incisors (***p*** < 0.01), versus round incisors in the opinion of restorative dentists (***p*** ≤ 0.03). The principles of visagism hold that female tooth shape is often more ovoid and delicate, while males are characterized by a more square tooth shape associated with virility [48].

In view of the controversial results, the selection of tooth shape based on gender lacks adequate scientific support and reliability. In the case of doubt, Nold et al. recommend selecting central incisors with an oval shape, as there is a greater probability of correlation with the natural teeth than when other tooth shapes are considered, independently of patient gender [33].

With regard to smile width, our results indicate a greater prevalence of exposure to the second premolar (61.4%), in agreement with the observations of other authors [29, 31]. However, Tjan and Miller [19] found the highest prevalence to correspond to exposure to the first premolar (48.6%). A possible explanation for this discrepancy is that these authors analyzed a sample (North Americans aged between 20 and 30 years) different from our own. Moreover, they found that only 4% of the subjects exposed their upper first molars when smiling – this finding being clearly different from the 20% recorded in our series. Aesthetic restoration of the visible posterior teeth may prove necessary in order to secure a harmonious outcome [49]. This is an important factor when planning anterior restorations, since consideration is required of both smile width and the number of teeth exposed. Variations in smile width allow for a broad range of situations considered to be aesthetically agreeable [50].

The results obtained in relation to the curve of the upper lip indicate a predominance of high lip curves (47%). Similar findings have been made in other studies such as that published by Hulsey [51], though in contrast Nold et al. [33] and Liang et al. [21] recorded a greater frequency of straightened curves. The differences with respect to Liang et al. [21] are probably due to the fact that their sample consisted of Chinese individuals, and ethnicity may play an important role in this regard.

## Conclusions

The different aesthetic parameters must be efficiently integrated in clinical practice, since a range of disciplines are implicated in patient management (restoration, prostheses, orthodontics, periodontics). No significant gender differences were observed in relation to the parameters studied, with the exception of the smile line, which was found to be higher in females than in males. The population studied has a maxillary interincisal midline centered with the facial midline, a consonant arc of the smile, an upward lip curve, a medium smile line, with exposure to the second premolar, and an oval tooth shape. The present study may serve as a guide for dental professionals in orthodontics, restorative dentistry or prosthetic rehabilitation, among other fields.

## Data Availability

The datasets used in this study are available from the corresponding author on reasonable request.

## Abbreviations

ArS: Arch of the smile
CL: Curve of the lip
Fis: Fisher test
FML: Facialmidline
IML: Maxillary Interincisal mildilne
MW: Mann-Whitney test
Sh: Shape of the teeth
SL: Smile line
WS: Width of the smile
